# The Hospital. Nursing Section

**Published:** 1905-10-21

**Authors:** 


### The Hospital.

### Nursing Section.

Contributions for this Section of "The Hospital" should be addressed to the Editor, "The Hospital" Nursing Section, 28 & 29 Southampton Street, Strand, London, W.C.

No. 995.—Vol. XXXIX. SATURDAY, OCTOBER 21, 1905.

### Notes on News from the Nursing World.

### THE QUEEN AND HER NURSES.

We must confess that the particulars which we are able to give in another column respecting the Nurses' Home at the Millbank Military Hospital justifies the impression that the building is likely to fall far short of what it ought to be. The fact is that the Nurses' Home at the army headquarters should be a model Home, and thus constitute a considerable attraction to nurses entering the Service. How this can be possible so long as the sleeping accommodation consists of a large ward partitioned off into small bedrooms containing no fireplaces, while the sisters and staff nurses are to have a common sitting-room, it is difficult to imagine. Happily, however, the last word has not been said on the subject, and we cannot but believe that the improvements in the plans which the Queen, as head of the Imperial Military Nursing Service has a right to ask for, will be made at any rate to the extent of adding another floor to the building as it stands. Although nothing, we fear, would now render it quite an ideal Home, the addition would materially increase the comfort of the nursing staff, which is so evidently the object that Her Majesty has at heart. The nation will expect her wishes to be carried into effect.

### PROMOTIONS AT CHARING CROSS HOSPITAL.

We are officially informed that at the meeting last week of the Council of Charing Cross Hospital, Nurse Lilian Hollings was appointed out-patients' sister; Nurse Maud Hopton, sister to the Robertson Ward, men's medical; and Nurse Grace Hamilton, sister to the George Drummond floor, men's and women's surgical. All these nurses received their training at Charing Cross Hospital and have thus been promoted from within to the rank of sister—an admirable precedent, which we hope will be followed elsewhere.

### THE CASE OF NURSES AT SMALL INFIRMARIES.

The interest excited at the South-Western Poor-law Conference by the paper of the Matron of Birmingham Infirmary is a compensation to Miss Gibson for the time she must have devoted to her task and for her journey from the metropolis of the Midlands to Plymouth. A summary of her paper and the discussion which followed appears in another column. Not less valuable than her three practical suggestions for the better administration of Union infirmaries, was the sympathy she expressed with young women fresh from their training schools, often with luxurious nurses' homes, who find themselves alone in a small country Union, without any society, and no one with whom difficulties can be talked over. Coming from the head of a great and successful training school like Birmingham Infirmary, Miss Gibson's recognition of the drawbacks under which they labour should have an encouraging and stimulating effect. On this point one of the delegates suggested that ladies in the neighbourhood of the Union could do much, not only to cheer the inmates, but also to brighten the lives of the nurses, by becoming acquainted with the former and making it pleasant for the latter when they are off duty. This might work well in cases where the ladies are liberally endowed with tact, and the nurses capable of appreciating the courtesy.

### IMPORTANT CONFERENCE AT MELBOURNE.

A meeting full of importance to the nursing profession took place in the Melbourne Town Hall on September 7. The Royal Victorian Trained Nurses' Association recently issued a circular to all the public hopitals in the State setting forth the regulations which it desired should be adopted, with reference to the training of nurses, and post-graduate training of matrons. About thirty hospitals were represented and the Conference proved how thoroughly in earnest these hospital managers are. After a great deal of discussion the State School certificate of merit, or its equivalent, was accepted as the minimum standard of education for probationers. The post-graduate course for matrons was accepted in its entirety with the exception of the Obstetric Nursing Certificate. All agreed as to the usefulness of this knowledge; but objection was taken to it being made a compulsory subject. A nurse, in order now to qualify for the position of matron, must take in addition to three years' general training, infectious diseases nursing. She must also have had twelve months' experience in some responsible position, such as staff-nurse, or ward sister in a recognised training school. She must likewise pass in dietetics, household economy, hospital management, organisation and equipment. This will in the near future insure better teaching and more economical management in Australasian institutions than has obtained up to the present time.

### STALL-HOLDERS IN NURSING ATTIRE.

Speaking on Tuesday at the opening of a bazaar at Blackpool in aid of the Victoria Hospital, Lord Derby said, "I am told that pretty nurses are an advantage in a ward. They make the patients cheerful." The pleasantry is pardonable. But we think that Lord Derby would have been much nearer the mark if he had said that cheerful nurses make the patients cheerful. A nurse who is merely pretty may exercise a depressing influence in the wards. We suppose that the reason the stall-holders at the Blackpool bazaar were attired as nurses was because the promoters thought that the costumes would look pretty.

### STOKE-ON-TRENT WORKHOUSE HOSPITAL.

There is a movement on foot to separate the Stoke-upon-Trent Union Workhouse, from the workhouse hospital, and thus relieve the master and matron of the workhouse from any responsibility as regards the latter. As the workhouse holds upwards of a thousand inmates, of which nearly 400 are in the hospital or lunacy wards, the separation is most desirable from a nursing point of view. We hope that the result of an inquiry by a committee will be to carry the proposal into effect. Under present conditions it is obvious that the nurses must suffer from disadvantages in the discharge of their duties which ought not to exist in such an important institution.

### THE NURSING AT MONSALL HOSPITAL.

At an inquest held last week by the Manchester City coroner on the body of a commercial traveller who died in Monsall Hospital, some serious charges were made against the authorities by the friends of the deceased. It was alleged that he did not receive the attention in the way of nursing which he ought to have had, and that the nurse was withdrawn at a critical period. The Medical Superintendent of the hospital, however, who saw the patient at least once every day, stated that "there was no reason whatever for the complaint as to the nursing attention he received," and that he was "quite satisfied that all his directions were properly carried out." The coroner having also affirmed his conviction that the deceased had the best medical aid that could be provided in Manchester, the best possible accommodation, and adequate nursing attention, the jury found that death was due to natural causes and declined to endorse the complaints against the hospital.

### THE HARROW HOSPITAL.

It is interesting to learn that the general plan of the enlargement of the Harrow Hospital was the idea of Miss Peake, matron of the institution, the architect, Mr. Arnold Mitchell, having put it into form. The co-operation of matrons in such cases is cordially to be welcomed, if only because they are able to advise on practical and technical arrangements, and thus prevent the mistakes which are almost unavoidable unless the architect has had considerable experience in hospital construction. We happen to know that the matron of one of the largest London hospitals now in course of erection has been closely associated with the planning of the new institution.

### THE VALUE OF PRESENCE OF MIND.

At Burslem Sanatorium last week a nurse was standing near the fire when her dress ignited, and she was speedily enveloped in flames. As no one was near to assist her, she rushed upstairs to the bath-room, turned on the water, jumped in the bath, and soon extinguished the flames. She was badly burnt, but is making favourable progress. To her presence of mind she doubtless owes the preservation of her own life, but it seems to us a great pity that every one is not taught that quick movement tends to spread the flames, and therefore involves considerable risk.

### MASSAGE AT GUY'S HOSPITAL.

A Massage Department in connection with the Actino-therapeutic and Electrical Department has been opened at Guy's Hospital, in which massage treatment and exercises are given daily. The number of cases under treatment are between fifty and sixty. The autumn work has been arranged and the probationers' medical lectures and classes, also the massage classes, have commenced.

### DANCES AT CAMBERWELL INFIRMARY.

An "At Home" was given the other evening at Camberwell Union Infirmary to the nurses by the matron upon her return from her holidays. Dancing took place from 9.30 to 11.30, many of the nurses being attired in fancy costume. "Two little nigger girls," "Chinaman," "Arry and Arriet," "Boy Blue," a "Tennis Player" (by one of the sisters in flannels), an "Early Victorian Lady," and other characters clad in all varieties of colour were represented. A dance now takes place at the infirmary every other Thursday after the late lecture, to which the medical men are invited.

### NORTHAMPTON NURSES AND THE PENSION FUND.

At the October meeting of the executive committee of the Northampton Town and County Nursing Institute, a scheme for federation with the Royal National Pension Fund for Nurses was unanimously adopted. The secretary of the Fund recently visited the institute, and, in an interesting address to the nursing staff, gave weighty reasons why nurses should secure policies.

### SAD DEATH AT POPLAR HOSPITAL FOR ACCIDENTS.

An investigation was held last week concerning the death of Miss Jessie Gertrude Southwell, a probationer at the Poplar Hospital for Accidents, who had just received her certificate of training. The matron of the hospital, in her evidence in the Coroner's Court at Stepney, said that Miss Southwell had been ailing since August, and in a nervous condition. She was unable to sleep, and was given a sleeping draught on Wednesday night. She was informed the next morning that the deceased had fallen out of a window in the top floor passage to the ground, a distance of sixty feet. The certificate, she added, had been granted to Miss Southwell a short time before it was due, in order to relieve her anxiety. When found she was fully dressed, except her cap and shoes. The jury returned a verdict that the evidence failed to prove the circumstances under which the deceased met her death, but we are afraid that she did not possess the mental stability which is an indispensable qualification for a nurse.

### A MAGNIFICENT RESULT.

A floral bazaar which has just been held in the New Town Hall at Walsall, in aid of the funds of the Victoria Nursing Institution realised no less than a total of £2,283. This is the more remarkable as the promoters only asked for £500 with which to clear off a debt, and a further sum of £1,000 to augment the endowment fund. The bazaar lasted five days, but the honorary officers were so delighted with the magnificent result that they invited the stall-holders and workers to a Cinderella dance on the subsequent Monday, and many congratulations were offered. Miss Holloway, the manager of the institution, was presented with a handsome dressing bag during the evening by the stall-keepers and helpers.

### THE NEW SUPERINTENDENT NURSE AT LEAVESDEN ASYLUM.

The excellent traditions of Leavesden Asylum have been observed by the choice of the new superintendent nurse. Miss Alice McLaren, whose appointment was sanctioned by the sub-committee of the Metropolitan Asylums Board last week, was trained at Glasgow Royal Infirmary, where she afterwards acted as staff nurse and assistant matron, has been private nurse at a well-known nursing institute in the South of England, was for upwards of three years night superintendent at Birmingham Union Infirmary, and for the last two years and a half has been assistant matron at Stirling District Asylum. Her experience, therefore, covers a very wide ground, and she will take up her important post at King's Langley thoroughly well-equipped in practical knowledge of both general and mental nursing.

### THE IRISH NATIONAL FUND FOR NURSES.

At a meeting of the Council of "King Edward the Seventh's Coronation National Fund for Nurses," held in Dublin last week, the report of the honorary treasurer for the quarter ending October 1 was received, and a direction given for the investment of £100 to add to capital, a portion of the balance in current account. Applications from eight nurses for membership were considered and six accepted.

### THE AMERICAN SCHOOL NURSE.

In 1902, at the suggestion of Miss Wald, the head of the Nurses' Settlement, the New York Departments of Health and of Education agreed to attach trained nurses to some of the schools, to see that the orders of the "school doctor were properly attended to." As a result, every school is visited by the Board of Health nurse; but her efforts are as yet more or less confined to caring for the child in cases of contagious diseases or when afflicted with eye troubles. But there is a suggestion that a further development should be made with regard to the school nurse, and that she should become "the State's most indispensable agent in keeping down its budget for its foundling asylums, hospitals, juvenile courts, truant schools, and reformatories," and "a potent means of solving the problem of the underfed or neglected child." In order to render her fit for such a position it is proposed that the school nurse shall have something more and something less than the usual training of the professional nurse; in short, a special social training, and perhaps dispense with part of the medical training. To accomplish this, a writer in the organ of the New York Charity Organisation Society thinks that the American School of Philanthropy and a maternity hospital school, by uniting, might supply the needful curriculum. The outcome will be awaited with interest on this side of the Atlantic.

### BEQUEST TO A NURSE.

The late Colonel Henry Coker Adams, of Anstey Lodge, near Coventry, who died on August 30, left a legacy of £100 to Miss Margaret Elsie Dust, of the Coventry and Warwickshire Hospital. Colonel Adams in the early part of last year met with a severe accident, and was removed to the hospital, where he remained for three months. Upon leaving the institution he gave a cheque for £800 towards the provision of a fund for the building of a "Nurses' Home," and his subsequent kindly recognition of Nurse Dust shows that the services rendered to him at the hospital were greatly appreciated.

### IRISH MATRONS' ASSOCIATION.

With reference to the note last week headed "Irish Nurses and Massage," there has been some confusion on the part of our correspondent between the Irish Matrons' Association and the Irish Nurses' Association; there is no matrons' council in Dublin. Classes for instruction in massage, the hon. secretary of the Association informs us, are held under its auspices by Irish teachers who were trained in Dublin for the Incorporated Society of Masseuses' Examination.

### WHY PARIS CANNOT ADOPT THE ENGLISH SYSTEM.

Although the English system of nursing has been introduced with so much success in Bordeaux, it has not yet been adopted in the Paris hospitals. Improvements have been made in many of the institutions in the French capital, and the Sisters of Charity who were working in the name of a "Confession" without the slightest professional knowledge have been removed. Schools for "Gardes-malades" have also been established; but we are told that Parisians are "not rich enough to adopt the liberal system used in England."

### SHORT ITEMS.

The Bethnal Green Guardians have increased the salary of the matron of the infirmary from £100 to £110 per annum, with further annual increments until a maximum of £150 is reached.—Miss F. N. Learmouth, of the Army Nursing Service Reserve, sailed on Saturday for duty in South Africa.—Mrs. Herbert Campbell has resigned her position as lady superintendent of the Liverpool nursing division of the St. John Ambulance Brigade in consequence of ill-health. The value of her services has been warmly acknowledged.—St. Thomas (Exeter) Guardians have decided to subscribe £5 per annum towards the Otterton District Nurse, on condition that she attends the old people in her district free of charge.—The name of the gentleman whom Miss Wilson, formerly district nurse at Harrow-on-the-Hill married the other day, is Mr. Ritt Bonarjee. Mr. Bonarjee is well known in India.

### The Nursing Outlook.

" From magnanimity, all fear above;
From nobler recompense, above applause,
Which owes to man's short outlook all its charm."

### ORGANISATION BY COUNTIES.

With a view to promote and expedite the efficient organisation of nursing in all its branches in every part of the United Kingdom it is desirable that a complete scheme should be prepared for each county. Such an organisation is essential for perfecting the machinery and arrangements necessary to fulfil the requirements of nursing in its twofold aspect. Few people realise that supply and demand in nursing matters necessarily mean supply and demand in regard to the raw material, as well as to the completed article. No doubt, as the Parliamentary Committee indicate, the nursing homes and agencies which make a profit out of supplying nurses to the public, should be registered and placed under the direct control of the local authorities everywhere. They are largely responsible for the existence of the pseudo-nurse. They are the fosterers of the relatively incapable woman, who claims that she can always earn good money as things are at present, and that there is no need for her to attend post-graduate classes, or to enter for a qualifying examination should the opportunity be given her. So long as the supply agencies are unregistered, so long will the pseudo-nurse be able to maintain this position. Once register and control the sources of supply of nurses to the public, and the pseudo-nurse will be compelled to obtain a qualifying certificate, or to abandon nursing for some other pursuit, if she wishes to support herself by her labour. Each municipality and county should lose no time in taking the necessary steps to empower the authorities to register and control nursing homes and agencies for the supply of nurses.

The county organisation must however include the training schools and unite them and all other institutions possessing material for the training of nurses, so that the maximum facilities for the adequate training of a sufficient number of nurses may be brought into action and kept efficient. Each county contains, in addition to the larger hospitals, at least one fever hospital, one lunatic asylum, a poor-law infirmary or sick wards, and several of the smaller special hospitals and institutions of various kinds, which, as they may all contain valuable material, should be co-ordinated and brought into co-operation for the purposes of nurse training.

It is desirable that the county association should include representatives of any district nursing association working within its area, and of any other considerable agency managed by a committee which may supply nurses to practitioners or the public. The central idea of any scheme which can prove adequate and satisfactory for the purposes here suggested should be to bring the whole of the nursing arrangements of the county under the general supervision of the county association, the council of which should consist mainly of representatives of the institutions which train probationers and employ nurses. The council might consist for instance of twenty-one members, a third of whom might be representatives of the training schools, a third members of the medical profession, and the remaining third representatives of the public who employ nurses and provide the funds to run the hospitals and kindred institutions. Such a council, if care be taken to secure that the representatives of the different sections are in fact the best and most trusted members of the community, should readily formulate a scheme whereby the probationers employed in each institution were engaged upon identical conditions, or nearly so, and were privileged to undergo a complete and thorough training in every branch of nursing, the curriculum being so designed as to apportion the time in the most economical and successful way.

Each county association must necessarily consider initially whether they will themselves establish a central home and organise a system of lectures and instruction in the preliminary subjects in which probationers must qualify, or whether they will try other means of securing the same end. There can be no question that the right course for each county association to follow is to establish a central home where the preliminary duty of selecting applicants who offer themselves for instruction shall be undertaken, and where those who may be selected shall undergo the preliminary tests and training before they are passed on to the hospitals.

By undertaking this work the associations will be provided with a definite income derived from the payments which the candidates will make during their residence on probation at the central home. They will in this way confer considerable assistance upon the chief officials of the hospitals and kindred institutions where the probationers will ultimately go for practical training, and at the same time be in a position to make arrangements with the general hospitals for the major portion of the training, and with the fever and other special hospitals for the courses which each probationer will pursue at these latter institutions during some portion of the period of her training. In this way the central association will be able to organise effectually for the utilisation of all the material within the county. We are prepared and shall be very happy to render every assistance in our power to help forward this movement, and we invite correspondence upon it.

### Laryngeal Diphtheria.

By Mrs. HICKS, Formerly Matron of the Evelina Hospital for Sick Children.

### I. INTUBATION AND TRACHEOTOMY.

One of the most important points with regard to the nursing of diphtheria is the care which must be taken to prevent the infection spreading, as it is a highly contagious disease. The patients should be nursed in a special ward, and the nurse who looks after them forbidden to enter any other ward. The infection, being carried by the expectoration, saliva, nasal and other discharges, it stands to reason that these excretions and secretions should be rendered harmless as soon as they are within reach. The pieces of soft linen or muslin with which the discharge is wiped away should be thrown into a bowl containing a solution of carbolic acid 1-20 and burnt as soon as the nurse can leave the patient's bedside. Her hands should be well rinsed in a 2 per cent. solution of lysol whenever they have come in contact with the patient, the bed, or anything he has used. The nurse should remember never to touch her face or any part of her body or uniform before this disinfection has taken place. The nurse's sleeve should be made so that the part below the elbow is detachable; and I recommend the diphtheria nurse to leave these undersleeves outside the ward altogether, and thus avoid the risk of bringing her cuffs into contact with the patient. It is impossible to attend to a patient in times of great anxiety, trying to hold him down when struggling, without touching the bed with the underpart of the arm. When this is not covered by a sleeve there is no harm in the contact, because it is as easy to soak the bare arm as the hands.

All the linen, utensils, crockery, etc., for the diphtheria ward should be distinct from those used elsewhere, for example—marked with a large blue cross so that they cannot possibly be mixed up with those of other wards. In addition, it should be remembered that no food, which has been in the ward, must leave it; all remains must be burnt.

Here, alas! it is necessary to be extravagant; but this waste can be reduced to a minimum by only taking into the ward what is absolutely indispensable. Where there is a sink in the ward the nurses can wash their own crockery, but they should always remember to soak everything in disinfectants before washing it, so as to avoid admitting infection into the drains. The contents of the bedpan should be mixed with an equal quantity of carbolic acid lotion 1-20, before being taken to the lavatory, where a special mop should be kept standing in a blue-crossed jar of 1-20 carbolic lotion. In an infectious ward the nurse will grasp better than anywhere else the necessity of dusting with a damp duster, and it should be wetted with carbolic lotion instead of with water, and the duster left soaking when the ward work is finished. The dirty linen should be kept in covered pails, which should be emptied into the foul-linen washer twice a day, the pails afterwards being scrubbed inside and out with formalin solution. When the diphtheria nurse goes to her meals she should thoroughly disinfect her hands, take off her apron, and outside the ward put on her lower sleeves and a clean apron. In addition she should gargle her throat with potassium chlorate gargle before every meal.

After these preliminary remarks I will go through the general routine of admitting and nursing the patient. The nurse should, to begin with, try and remember that the majority of doctors like their diphtheria cases nursed flat, and therefore make it a rule always to do this, unless they give orders to the contrary. For a new patient who may be struggling, straps are as a rule necessary, and as these allow of plenty of movement, excluding only the sitting-up posture, the child soon gets accustomed to them. As soon as possible save a specimen of the child's urine. Albuminuria is a frequent complication of diphtheria. A specimen can easily be obtained, as a rule, while taking the child's temperature. The respiration of the patient must be watched with the greatest care. The nurse can, by observing the progressive symptoms attentively, be of great help to her patient, sending for the doctor at the right time. The child may be, or gradually becomes cyanosed, the lips blue. On uncovering the chest, the nurse may find the intercostal spaces being drawn in during inspiration, and the part below the ensiform cartilage almost sucked to the spine. The child will in this case be excessively restless, throw himself about violently, fighting with all his might for breath, with perspiration breaking out over the face; occasionally there will be the typical hoarse, stridulous, almost whistling cough. The only aggravation of this condition is when the child, gradually from exhaustion, gives up struggling and lies back almost apathetically, only emitting short sighing gasps, each one leaving the patient more livid. Of course the nurse must not wait for this condition to supervene to send for help. The recession of the intercostal spaces is the first indication of danger, and when it sets in, she should ring her bell, and others will judge whether interference is necessary. The glands of the neck are generally swollen in cases of laryngeal diphtheria.

A curtain for the bed and a steam-kettle are standing orders for cases of laryngeal diphtheria. Often, when the child is admitted with a fair but not excessive amount of stridor, a hot bath of about 104 deg. is ordered and does good in some cases. To a great extent the benefit of the bath depends upon the way in which it is given, and I may again point out to nurses that they must not frighten the child by plumping it straight into the bath. It should be put in playing, gently, cheerfully, as if it were being given a treat. In this breathless condition any abrupt movement and sudden change will aggravate the difficulties of the child, so the nurse should be gentle, sympathetic, and wise. Some doctors order vinum ipecac., and in this case the child should be allowed to drink one drachm of the drug with an ounce of hot water every ten minutes, until it has been sick. The nurse should then examine the vomit very carefully for any pieces of membrane. The membrane can be recognised by placing the suspected portion into a saucer with some water, and trying to pull it apart with two needles; ordinary mucous—which is often mistaken for membrane—will be divided by the needles, whereas the membrane, which is of a leathery consistency, will not come apart. All pieces of membrane must be kept for the doctor's inspection in a sterilised test tube, the mouth of which should be plugged with a piece of sterilised wool. If a doctor wishes to take a cultivation from the child's throat, he will bring his own mounted swabs, platinum needle, and tubes; the only things the nurse will have to get ready are: a tongue depressor, matches and a spirit lamp with which to sterilise the platinum wire and burn the cotton wool plug for the tube.

The wonderful results which have been attained since the introduction of diphtheria antitoxin in the treatment of this hitherto so often fatal disease render it most probable that antitoxin will be the doctor's first prescription for the patient, and hence everything should be got ready, so as not to retard the benefit of this grand treatment for a minute. The first thing to be done, when the nurse hears that she is to have a case of diphtheria, would be to place her antitoxin syringe and a small medicine glass in the sterilizer with cold water and light the gas underneath. It is desirable to use a Luer's syringe for injecting, as it is made entirely of glass and can be easily disinfected, the only precaution being not to drop it into boiling water, for obvious reasons. The other requisites would be those for disinfecting the patient's skin; some collodium, and either dry, sterile gauze or sterile absorbent wool. The sites for injection are those parts of the body where the skin can be easily lifted off, so that the underlying tissues can accommodate the fluid. Either groin or axilla is generally chosen, and disinfected in the usual manner, by rubbing with soap and water, ether and lot. hyd perchl. 1-2000. By this time the syringe should have been boiled and allowed to cool, and the antitoxin bottle—which has been carefully opened and its mouth wiped with sterile wool—either emptied into the medicine-glass and sucked up, or poured straight into the syringe by the doctor, who will then inject the drug very gently, the nurse doing her utmost to immobilise the child, so that the needle does not stray. The small opening should then be covered with wool or gauze and collodium.

One word about antitoxin. Every hospital should keep a small supply, not much, because it easily spoils; it must be stored in a cold place, but not allowed to freeze. Antitoxin is sold in small bottles containing doses of various strengths. This is not estimated by fluid or weight measure, like other drugs, but by the amount of remedial power it contains. The minimum of this remedial agent is called a unit, and phials of antitoxin are sold containing from 500 to 4,000 units. The doctors generally begin with an injection of 2,000 units (about four c.c), which dose is often repeated after 12 or 24 hours, when it becomes necessary to purify the opposite side of the part, which has been previously prepared. The whole contents of the bottle are, as a rule, used; but, should any of the fluid be left, it must be thrown away, as it has lost its sterility through contact with the air.

If the patient is taken in time, the antitoxin will soon do its work and the membrane, which may have draped the soft palate and tonsils, will, within 24 to 36 hours, have almost entirely dissolved, and the patient will soon recover. Since the introduction of antitoxin there has been little resort to local treatment, most physicians trusting to the drug alone. If swabbing of the throat is ordered, the ordinary sponge-holder should be used, with a ball of wool wrung out of the paint or lotion prescribed. Press down the tongue, holding the spatula in the left hand, and with the right, swab out the throat thoroughly, but gently. The nurse should examine the swab for membrane before burning it, and be careful not to put her face directly opposite the child's, else it might easily cough some of the discharge into her face; if this should happen, she should quickly bathe it with antiseptic solution, and the eyes with sol. hyd. perchl. 1-4000. She should try to prevent the child from rubbing any expectoration over its face which might carry infection to the eyes. The complications which occur most frequently are kidney and lung trouble. All that nurses can do to try and prevent these is to keep the child warm and clean in a well-ventilated room, and save the 24-hour specimen of urine daily. By far the most dreaded foe, however, is heart failure. To avoid this the patient should be kept absolutely quiet, rigidly preventing any sudden movements, excluding even such excitements as parents' visits. These are only allowed in extreme cases, and then preferably when the child is asleep.

The diet ordered generally consists of milk, milk puddings, eggs, and beef juice; when albumen is present in the urine the latter is, as a rule, left out. If brandy is prescribed the nurse should be careful to give it well diluted, and slowly, so as to avoid choking. She should be ever prepared for the dreaded collapse and have all her hypodermic solutions and syringes in perfect order, so as to be ready at a second's notice. If the patient progresses favourably he will be practically well in a week, but the doctors usually keep him flat for three to four weeks, and then very gradually allow sitting up and walking. As the diphtheria bacilli are often found in a patient's mouth and throat and nose for months after the illness, it is well to warn the mother of the possible danger of kissing when she takes the child home, and also encourage her to let the child use the mouth wash which the doctor will order for a long time.

Another sequel of diphtheria which may supervene while the child is still in the hospital is post-diphtheritic paralysis. The early symptoms are, as a rule, a nasal tone of the voice (this being due to paralysis of the soft palate) the liquid food will regurgitate through the nose, and the child choke while drinking. Later on the paralysis may spread to the extremities, the eyes and the diaphragm. Your duty thus will be to report at once any change in the voice, regurgitation of the food, or the occurrence of a squint. The nasal tube will most likely be substituted for the mouth feeding, and the doctor will by further means try to overcome the symptoms.

Overalls for the use of the doctors, relieving nurses, and parents, are usually kept in a cupboard in the passage outside the ward, and these the parents must be asked to wear, having tucked up their skirts and their sleeves. Parents must disinfect their hands and faces before they leave.

We have so far spoken of cases that respond to antitoxin and improve, but if instead of improving the symptoms of laryngeal obstruction are aggravated and the dyspnœa increases, the doctor will have to perform one of two operations to relieve the patient—namely, intubation or tracheotomy.

As a nurse, it is not my place to speak in favour of one or the other method, but as an onlooker one cannot refrain from expressing the greatest admiration for the absolutely beautiful results I have seen with intubation during the first six or seven years after the introduction of antitoxin. To see a child blue and gasping for breath suddenly, in a few seconds after the introduction of the tube, take deep, regular respirations, regain its natural colour, and drop into a quiet peaceful sleep, is sufficient to fill the heart of any true nurse with a most grateful joy. If after two or three days the tube is removed and the child can breathe naturally, we are still further inclined to sing the praise of the simple method of relieving the dear little patient.

(*To be continued.*)

### The Nurses' Clinic.

### HOME-MADE BANDAGES.

This, of course, is no new suggestion for the trained nurse, but it may be found useful to some readers of The Hospital who do occasional nursing amongst the poor.

To the charitably inclined, the bandage item is a considerable expense with which to provide patients. Unless an aseptic bandage is required for some special case, bandages rolled *chez-nous* with a little trouble answer the ordinary purpose very well, and are, of course, much cheaper.

This is how I do mine. I buy one dozen yards of unbleached calico, which costs 2d. a yard. This I cut into two pieces of six yards each, tear off the selvedge, soak in hot water, and rub pretty hard to get the dressing out. Then I let the winds of heaven sweeten it in the garden, after which I iron and roll the whole piece; the ironing process is hardly necessary, but makes it nicer. Next I unwind a portion of the roll and cut small slits—mostly two inches, a few three, and one six. Next I take in my

**THE NURSES' CLINIC—*Continued.***

hand each alternate slit; someone else takes the remaining ones, and we pull against each other, tug-of-war fashion, until the end of the six yards is reached. Thus, in a few minutes, the whole is divided and ready for winding.

I put them all in a basket, and wind them roughly first, during which time I either read or listen to the man-about-the-house, recounting the wonders which surgery (occasionally man himself) achieves. Thus the monotonous part of the task becomes pleasant.

The bandages can be wound fairly tightly and evenly by hand, but a winder is certainly preferable; and a simple, practical one is on sale for 2s. 9d. This, of course, turns out a really good bandage, and by the time the end has been turned in V-shaped and a pin inserted, looks quite professional.

A one-inch bandage is not so easy to wind, so I take the six-inch I mentioned, sharpen the carving-knife, and after a few fell swoops, convert the whole into six neat finger-bandages. When bandaging I generally put on a turn or two more than necessary (except, of course, for eye or head cases); then, if the dressing comes through, I can cut off the soiled part and wash the rest. Or where the bandage is exposed and gets dirty, but the wound does not require redressing, the exterior twist can so easily be removed, leaving a nice clean one underneath to be revealed.

Old linen, of course, makes nice soft bandages which might be jealously guarded for baby patients; unbleached calico is too hard for soft baby flesh.

My bandages all finished, I arrange them in a deep, long wooden box, and keep them in a cupboard with a jar of pot-pourri. This last proceeding is, of course, an old maid's fad; perhaps a level-headed professional nurse would consider it an act of folly. But for all that, the doctor says my bandages are "really awfully nice," though, being "mere man," he does not know the reason; and neither would you if I had to sign my name hereto.

### A New Departure in the Training of Midwives.

BY A CORRESPONDENT.

The authorities of the Royal Maternity Charity, whose work amongst poor married women in their own homes has been going on for the last 150 years, are determined to move with the times, and have started a new institute for the training of midwives. As yet the scheme is in its infancy, but Mrs. Macdonald, the head matron and midwife of the Charity, was able to give me some interesting particulars of the new departure when I called upon her at 7 Delamere Terrace, Paddington. "We have room for twelve probationers in this institute," she said, "but at present we are not quite full. Before admission candidates must produce a certificate of birth, showing that they will have attained the age of twenty-one by the time they have completed their training, and a second certificate of good health and moral character, and signed by a person of satisfactory standing, who has known the candidate for at least a year. The fees are a guinea upon application and then £22 10s. in advance for the full midwifery course, and £10 10s. (resident) for the monthly nursing course.

"These sums, I conclude, cover board and lodging?"

"Yes, but non-resident pupils may join if they wish, in which case the cost is £13 13s. for midwifery and £6 (non-resident) for monthly nursing instruction. Upon entering the institute all pupils have to sign an agreement that they will abide by its rules and faithfully carry out all orders given them by the physician and lecturer, the secretary, or the matron. Pupils also pay for such articles of uniform, professional equipment, books, etc., as they require, and for their own personal washing. But print dresses, aprons, etc., are sent to the Charity's laundry.

"The training extends over a period of twelve weeks, as laid down by the Central Midwives Board, and is both theoretical and practical by means of lectures from the physician-accoucheur and lecturer attached to the institute, and by accompanying me to cases in the district. I always take two probationers to a case with me. One delivers the mother and the other washes the baby. Also, of course, I give them any extra instruction or coaching they need at home. It is my great desire to give to this institute more of a 'home' atmosphere than is possible in the large lying-in hospitals, whilst insuring to each pupil the twenty practical cases required by the Central Midwives Board, and making every effort to enable the pupils to pass the qualifying examination of the Central Midwives Board with credit to themselves and their instructors."

"Do you guarantee a pass to your pupils?"

"No, that is impossible. The success of an examination must necessarily depend upon a pupil's own aptitude to learn and her earnest application to the course of instruction. But if a pupil be absent during part of the course through sickness or other unavoidable cause the fees paid are allowed to stand to her credit, and she may later on, when able, resume her course to complete the twelve weeks' training. Moreover, provided that a week's notice is given she will not be charged for her weekly expenses during her absence."

"You say that you insure the necessary twenty practical cases to a pupil. Is your work in Paddington amongst the poor large enough for that?"

"There are three other district midwives attached to the Royal Maternity Charity living near here, one at Notting Hill Gate, a second in Belgrave Road, and a third in Harrow Road. I may at any time send my probationers to them for the sake of extra experience, so that there is not the slightest difficulty about the practical work."

"You call in a doctor to assist if necessary, I suppose?"

"Decidedly, and by the rules of the Charity in a case of emergency instead of having to send some distance to a special surgeon on the staff, any medical man may be summoned, whoever is the nearest, and the Charity pays all expenses. Jewish midwives are provided for those who belong to that religion. All the mothers are visited till the babies are ten days old."

"Have you more pupils for midwifery or for monthly nursing?"

"Most come to train as midwives, and I always endeavour, if possible, to persuade a monthly nurse ultimately to become a midwife. It is so much safer for an attendant on a woman in her confinement to be fully qualified. I well remember feeling when attending cases as a monthly nurse that I did not know all I should like to, and that was why I followed maternity by midwifery training. I trained at Queen Charlotte's Hospital, where I ultimately became head midwife for about four years. During that time out of 1,300 cases only one patient was lost. I was afterwards attached to the out-patient department. I joined the Royal Maternity Charity in 1883."

"But you have done private practice as well?"

"Yes, I have worked for some of the best London doctors and in all parts of the country. It was owing to the good offices of one of them that Princess Christian selected me to go out to the German Empress. I was with her twenty-two years ago when Prince Eitel Friedrich—whose betrothal to the daughter of the Grand Duke of Oldenburg is just announced—was born, and again with the next Prince. I was unable to attend her with some of the other children, but nursed her with the last baby, Princess Victoria. The kindly consideration of the German Royal Family to me I shall never forget, and the Empress herself allows me to state that I have attended her on three occasions. She also permitted me to call my own daughter, born after I attended my royal patient, by the name of Victoria, after herself. I have photographs of the Imperial family, which were given to me by Her Majesty, and I naturally value them much."

"Does the inspector of midwives visit this Institute?"

"Oh, yes, about every two or three months, examining the bags, etc., the same as in any other house occupied by a midwife, and though I am as careful as is possible over all the points insisted upon by the Board, I am always glad she should come and ascertain officially that all is in order. You see, I have been training midwives for so many years that it is comparatively easy work to me. Amongst other pupils I trained a German baroness, and also a lady who was afterwards presented at Court. Neither of these ladies wished to practise, but they wished to be capable of assisting a mother if required. One had nearly lost her dearest friend through ignorance and so made up her mind she would learn for herself how things ought to be done."

### The Matron of Birmingham Infirmary at Plymouth.

The principal event at the South-Western Poor-law Conference at Plymouth on Thursday last week, over which Sir T. D. Acland presided, was the reading of a paper by Miss Gibson, Matron of Birmingham Infirmary, on "The Administration of Union Infirmaries."

Miss Gibson said that after many years of patient toil, the difficulty of arranging the best methods for the care of the sick inmates of a large Union appeared to have been overcome, given the enlightened and philanthropic guardian and the wide-minded and large-hearted official. But even now it was only beginning to be understood that it was necessary that sick inmates of the Workhouse should be cared for as well and as skilfully as the sick in the hospitals, and that this could be done easily at least in Unions where, by reason of numbers and environment, it was as possible to engage and to keep the trained nurse as any other official. It was not many years since the Poor-law nurse was considered by other nurses a very inferior sort of person indeed; but now the large infirmaries were as well staffed on the medical and nursing sides as any hospital, and the probationers compared very favourably as regarded intelligence, education, and social standing with those who applied to the ordinary general hospital. Their quarters were usually comfortable and well furnished, their food good, and the hours of rest and recreation sufficient, and they gained an all-round experience which as an equipment for a nursing life could not be beaten. One great factor in the difficulty of properly nursing small infirmaries was that the nurses at present available (though even those are available in far too small numbers) were quite unaccustomed to, and quite unprepared for, the conditions which they found. She would ask them, before going on, to keep in mind the cheerful and sociable conditions under which a nurse worked during her days of training, and to glance for a second at the numerous ways which opened up to her, when her certificate was once gained, for going on with her profession. The Jubilee Institute swallowed up an enormous number, and others were claimed by private nursing and Colonial nursing, a considerable number married, and some emigrated. Keeping all those counter-attractions in mind, let them consider what the union infirmaries offered, and under what conditions a nurse there worked. Many of those workhouses were quite in the country, far from any centre where any form of social life could be found. Probably the nurse on her arrival found comfortable quarters, but she also found that she lived quite alone. Instead of going off duty, and finding all her meals ready for her, having the rest and refreshment of a good, well-cooked meal, accompanied by the society of a number of other nurses, she had an old workhouse inmate, who knew nothing of cooking, who prepared something for her. She was discouraged at the lack of appliances, she had tow for poultices, and she was expected to use old rags. She keenly desired someone to sympathise with her and advise her what to do. Before she came there she could have found a dozen fellow-nurses to talk over her grievances, which, when she could find no one, became proportionally greater. By and by the solitude became unbearable; she became listless, and for her own sake and for the sake of the poor sick it was best she should give up. This was exactly what she had seen happen over and over again. She had thought a great deal on the subject, and could see only three ways by which any change could be made for the better in the administration of union infirmaries:—(1) By removing the sick altogether from the workhouse, and placing them in convenient centres; (2) by arranging with the cottage hospitals to take severe, and the district nurses less severe, cases in unions where the number of sick was very small; (3) by forming centres in each district where probationers would receive most of their training, being drafted for one or two years to a small union for a part of it. She considered the plan of nursing in small unions by engaging probationers was a most dangerous and unsatisfactory plan. In no union, however small, could there be, if any pretence was to be made of efficient nursing, fewer than three nurses, one on night duty and the other two to relieve each other on day duty. As regarded the removal of the sick from surrounding unions to a centre, there were disadvantages, she feared, that would outweigh the advantages. It would seem to be very difficult to remove all the sick, and centralisation, she feared, would end in some, if not many, of the infirm and bed-ridden being left in the union with even less skilled care and attention than they had now. The greatest drawback to centralisation would be the removal of the helpless old people from the immediately neighbourhood of their friends. The second alternative she had given was open to two very serious objections. It did not arrange for the care of the aged and infirm, who would be left in the infirmary, so that the necessity of nurses would still remain, and it put the responsibility for the care of the patients into the hands of persons who were not servants of the Guardians. It was suggested that by giving the nurses more time off duty, better food, and larger salaries, they would be induced to stay, but those considerations would only attract the kind of nurse who had better not be attracted. With regard to the third possibility—the drafting of nurses from the large infirmaries to the small—before it could become in the least practical they must cease to be parochial and become national. It would be necessary to divide the country into districts, and probably probationers would require to be bound to serve for four years instead of three. The difficulty in connection with the extra nurses' accommodation which would be required by those infirmaries which undertook to train for others might be well got over by the smaller unions, which reaped the advantage, paying some proportion of the cost. The first year, and possibly the second year, of probation would be spent in the central infirmary, the third at one of the small infirmaries, and the fourth in the central infirmary again, to pass the final examination for the certificate.

### THE MATRON OF BIRMINGHAM INFIRMARY AT PLYMOUTH—*continued.*

Some such plan, if it would not entirely remove the present impasse, at any rate would greatly relieve it. But it would require a greater cohesion among Boards of Guardians than existed to-day.

Miss Gibson resumed her seat amid general applause, and a discussion followed. A delegate from Chard complained that Miss Gibson's paper depicted a very black condition of things, which was scarcely fair. The lady Guardian from Wells Union regarded the views of Miss Gibson as excellent in every way, more especially the third plan proposed. The Newton Abbot representative, whilst thanking Miss Gibson for a paper which seemed to him to have been everything that it should be, considered that the great difficulty was that young people went in for nursing without sufficient enthusiasm. At Newton Abbott they had an excellent infirmary, and did everything they could to make the nurses comfortable; yet they were always leaving. The delegate from Totnes agreed with the third proposal of Miss Gibson, and said that at Totnes they had not had much difficulty with their nurses; they had, in addition to female nurses, one male nurse, and the scheme worked excellently. The Chairman of the Devonport Guardians thought that Miss Gibson had placed before them an ideal scheme, and described the system by which his Board secured an adequate supply of nurses and probationers for Devonport, "in which happy spot," he said, "they knew nothing of friction." A Plymouth representative considered Miss Gibson's suggestions rather Utopian, and said that all the improvements could not be undertaken unless the money was forthcoming. The delegate from Okehampton said that the more they could engage lady visitors to see something of the actual work in the infirmary wards, become acquainted with the patients, and make it pleasant for the nurses when they were not on duty, the better would they be off in respect to nurses, and the happier would be the circumstances of the workhouse.

The meeting closed with a vote of thanks by the President on behalf of the Conference, to Miss Gibson for having come so far and for having read such an extremely valuable paper.

### The Nurses' Home at the Millbank Military Hospital.

In view of the attention excited by certain statements as to the Nurses' Home at the Military Hospital, Millbank, a representative of The Hospital paid a visit to the institution this week in order to ascertain how the question is regarded on the spot. The general feeling at Millbank is distinctly pessimistic as to any probability of the existing state of affairs undergoing a change for the better. In the original plans no provision was made for a Nurses' Home, and when it became apparent that this omission must be rectified it was determined to utilise one of the blocks intended for wards for the sick soldiers. This block stands in the rear of that portion of the hospital which looks on to the road. At the back its outlook is on to small tenements; in the front it will be close to the barracks, which are ultimately to take the place of the present open space. As it faces east, and will be within a few yards of other high buildings, it is obvious that its inmates will not see much of the sun. According to the original plan the wards, built as if required for the soldiers, are then to be divided off by lath and plaster partitions into cubicles, to be called by courtesy small bedrooms, each ten feet square, for the use of the staff nurses and sisters.

Some months since when the King and Queen visited the hospital they asked to see the plans of the Home. At that time the structure was only just above the ground. The Queen, in particular, examined the places carefully. She remarked that the bedrooms were too small, and that they needed fireplaces, appealing to the architect, who was present, as to whether these two defects could not be remedied. He replied that he was afraid not, as it would be impossible to build a different or a larger building upon the foundations, which were already laid. The Queen then made another suggestion. "If the original foundations could not be disturbed," said Her Majesty, "why could not bow-windows be thrown out so as to secure for the nurses better light and better ventilation?"

On the occasion of Her Majesty's next visit the Home had begun to assume more important dimensions, but as far as can be judged by those at the hospital—there is only one door to the building, and that being always locked none of the officials is able to speak with certain knowledge of the interior—no alterations in the original plans have taken place. The Queen, however, in giving vent to a hope that the arrangements for the nurses' quarters would ultimately be more satisfactory, mentioned that not only had she written herself, but that the King had also expressed himself strongly on the matter. Her Majesty, of course, made no allusion to the 2,000*l.* which the *British Medical Journal* states she offered in order to increase the accommodation without augmenting the cost to the nation.

No more definite information as to the arrangements in the Home is obtainable, for the sufficient reason that nothing further about the building is known at the hospital. It has been asserted at different times that there will be accommodation for thirty nurses and for sixty-three nurses respectively; that the matron will be provided with a bed-sitting-room and with a suite of three rooms; but the only accommodation allotted to the staff nurses and sisters, besides their separate sleeping apartments, appears to be a general sitting-room and a common dining-room.

### Incidents in a Nurse's Life.

### HOLIDAY DUTY IN A CONVALESCENT HOME.

I had been asked by a lady superintendent to act as locum tenens during her summer holiday in a large Convalescent Home for Children, with an average of 70 patients. Upon my arrival the Superintendent said, "Don't be alarmed should any of the boys plan to run away during my absence. Such a thing happened here once, much to my distress. There were two of them, and they were not missed until fairly late in the day. Then I sent to the police station and drove myself into the country, making inquiries everywhere. After a couple of very anxious days had elapsed I found a woman had secreted them in her cottage." And she added, "I gave her a good scolding, as no one can imagine the fright and anxiety it cost us."

However, the days passed by and all went quietly, until one day one of the nurses overheard two boys arranging to run hom, and came at once to tell me. I determined, if possible, to prevent this in a somewhat novel way. We gave the boys plenty of of amusement, games at home, tea on the pier, etc., so that they forgot they had been homesick, and settled down quite comfortably and were even heard to confide to a chum that "The sea was better than the East End." One very sad event happened which made my heart ache. The rule was for the Lady Superintendent to see all the children immediately after their breakfast. One morning, upon entering the boys' playroom, I found one of them on the couch. He said "he had a sore throat." I isolated him at once, sending for the doctor, who diagnosed the case as diphtheria. He ordered him to be removed to the Isolation Hospital belonging to the Home—a lovely cottage in grounds overlooking the sea. The little lad got on fairly well, improving daily; but suddenly one day he was taken worse, and the doctor came in looking very sad, saying, "All is over, he has passed away." Meanwhile the poor mother had arrived, heart-broken, but her surprise was great at our kindness and sympathy. She apparently could not realise why we should be distressed, and when his body was removed and she found we had a wreath prepared, she could hardly express her gratitude. He was "the only son of his mother."

One night later I was called to the large dormitory, as the boys would not be quiet. Nurse said, "I can do nothing with them." I found them bolstering and having a game. Very quietly I told them they must be quiet and go to sleep, as they kept the whole landing awake. I left them, but remained quietly outside for fear I might be wanted again. From this point of vantage I overheard the ringleader say: "Them nurses think themselves mighty clever, but not one is as clever as my mother, for she can take her teeth in and out of her mouth, and I know not one of them can do that." The amusement when I told this to the nurses can be easily imagined. But there was no more disturbance that night.

### Appointments.

[No charge is made for announcements under this head, and we are always glad to receive and publish appointments. The information, to insure accuracy, should be sent from the nurses themselves, and we cannot undertake to correct official announcements which may happen to be inaccurate. It is essential that in all cases the school of training should be given.]

Barry Town Accident Hospital.—Miss Mary Evans has been appointed working matron, and Miss Annie Rogerson and Miss M. B. Ellingworth nurses. Miss Evans was trained at Gloucester General Infirmary, where she has since been sister in charge of wards and night superintendent. She was subsequently in charge of the City Isolation Hospital, Gloucester. Miss Rogerson was trained at North Shields Victoria Jubilee Infirmary, and has since been charge nurse at Boston Hospital and charge nurse at Monkwearmouth and Southwick Hospital, Sunderland. Miss Ellingworth was trained at the Purton Union Infirmary, has since done private nursing, and been nurse at the Montague Surgical Hospital for Nurses.

Cheltenham Union Infirmary.—Miss Florence L. Tudball has been appointed charge nurse. She was trained at the Stapleton Infirmary, Bristol, where she was afterwards staff nurse, and has since been staff nurse at Kettering General Hospital and Barry Accident Hospital. She has also been on the private staff of the Northamptonshire Nursing Institution. She holds the certificate of the Central Midwives Board.

Darley Dale Cottage Hospital.—Miss Bessie E. Edmondson has been appointed staff nurse. She was trained at Burnley Infirmary, Lancashire, and has since been night nurse at the Accident Hospital, Mansfield, Notts.

Leavesden Asylum, King's Langley.—Miss Alice McLaren has been appointed superintendent nurse. She was trained at Glasgow Royal Infirmary, where she was afterwards staff nurse. She has since been assistant matron in the same institution, private nurse at the Kent Nursing Institute, night superintendent at Birmingham Union Infirmary, matron at Clarence Lodge, Clapham, and assistant matron at Stirling District Asylum.

Macclesfield General Infirmary.—Miss Florence Yoxall has been appointed sister of children's wards. She was trained at the North Staffordshire Infirmary and Eye Hospital. She has since been staff nurse and sister in the same institution, and in charge at Millfield, Rustington.

Melton and Belvoir Isolation Hospital.—Miss Clara Shields has been appointed matron. She was trained at the Fever Hospital, New Cross, London, and has since been superintendent nurse under the Leicestershire Small-pox Hospital Committee.

Northampton Union Infirmary.—Miss E. M. Sheen has been appointed nurse. She was trained at the Moseley Children's Hospital, Birmingham, and has since been assistant nurse at the Infirmary, Swindon Road, Cheltenham.

Portsmouth Workhouse Infirmary.—Miss Louisa Williams has been appointed night superintendent and Miss Beatrice Lilley charge nurse. Miss Williams was trained at Poplar and Stepney Sick Asylum, where she has since been staff nurse and ward sister. Miss Lilley was trained at Portsmouth Workhouse Infirmary, and has since been nurse at the Military Hospital for Women at Aldershot.

St. George's Infirmary, Fulham Road.—Miss Agnes Welchman has been appointed sister. She was trained at Greenwich Union Infirmary, where she was subsequently staff nurse. She holds the certificate of the Central Midwives Board.

Shirley Schools, Wickham Road, Croydon.—Miss F. E. Capes has been appointed matron. She was trained at St. George's Infirmary, Fulham Road, London, and has since been head nurse at Sutton Schools, charge nurse at St. Olave's Infirmary, assistant matron at Ladywell Workhouse, matron at St. Olave's School, matron at St. Michael's Orphanage, and matron at Morley House, St. Margaret's Bay, near Dover.

West Ham Union Infirmary.—Miss Fanny Esther Barrow, Miss V. G. Godfray, and Miss M. Prudence have been appointed sisters. Miss Barrow was trained at the Poplar and Stepney Sick Asylum. She has since been staff nurse at the Throat and Ear Hospital, Brighton; on the private nursing staff of Brighton Nursing Home; temporary night nurse at the North West London General Hospital; and night sister in charge of Mount Vernon Hospital for Consumption, Northwood, Middlesex. Miss Godfray was trained at St. Mary's (Islington) Infirmary, where she has since been staff nurse. Miss Prudence was trained at St. Mary's (Islington) Infirmary, where she has since been staff nurse.

Wolverhampton and Staffordshire General Hospital.—Miss Ethel Kelland has been appointed sister of the children's and women's accident wards. She was trained at the Norfolk and Norwich General Hospital, and has since been sister at the Monsall Hospital, Manchester.

### Death in our Ranks.

Royal Berkshire Hospital.—We hear, with much regret, of the death of Miss Wise, who for upwards of seventeen years was a nurse at the Royal Berkshire Hospital, Reading, for some years in the wards of the Hospital, and more recently on the private nursing staff. She resigned her post this year in consequence of ill-health, and was staying at Knowl Hill Vicarage when acute symptoms of appendicitis developed, and she was removed in an ambulance to the Royal Berkshire Hospital, where an operation was successfully performed. Five days later, however, she succumbed, and her remains were laid to rest in Highmoor Churchyard, a memorial service being held in the chapel at the hospital.

### A Book and its Story.

### A DAUGHTER'S DIARY.

"The Household of Sir Thomas More," published by the de la More Press, is one of the series of "The King's Novels" which are being brought out under its direction, and is an admirable reprint of a little book written years ago by Anne Manning, a sister of Cardinal Manning's. There is a quaint charm about this chronicle of an English household at the momentous period in which Sir Thomas More lived. A period which stands in history at the parting of the ways when "mediæval civilisation was going out and modern civilisation was coming in." Historically the book has little value. Its chronology is at fault in introducing Erasmus, who at the time the Diary purports to be written was not in England, nor did he visit the country after Margaret Roper, the married daughter of Sir Thomas who kept the Diary, was born. By other inaccuracies the general reader will not, perhaps, be disturbed, but will find in the genial domestic picture drawn by the author of the distinguished statesman in his Chelsea home surrounded by his family full compensation for the exercise of occasional historical licence. Margaret Roper was her father's favourite daughter. She married William Roper, the first biographer of More. As is well known, Sir Thomas was the great statesman and judge who succeeded Cardinal Wolsey as Lord Chancellor, and from being the trusted friend and councillor of Henry the Eighth incurred the royal displeasure by opposing his marriage to Anne Boleyn and his divorce from Catherine of Arragon. More, after the resignation of his office, retired into private life in the then little village of Chelsea. The diary dates from this period until his execution for refusing to acknowledge the King as supreme head of the Church. He was a zealous adherent to the old faith, and his public and private life were exceptionably blameless. As a statesman and scholar he stands out as one of the most learned and distinguished Englishmen of his day. In his private character as father and friend we have, in the diary, a pleasant glimpse which has its value as accentuating the cordial relations that existed between himself and his family at a time when these relations were apt to be strained. He had naturally a distinguished circle of interesting and scholarly friends, but Miss Manning has preferred to dwell chiefly on his home life. Of the affectionate relations existing between parents and children the following extract written on her sixteenth birthday by Margaret will illustrate :—"Father away which made it sad. Mother gave me a pair of blue hose with silk clocks, Bess a bodkin for my hair, Mercy a saffron cake." After the enumeration of other gifts comes the entry of one from Will Roper, who was then only a diffident supplicant for the favours of his future wife. Throughout the Diary Margaret's remarks suggest that she has the stronger character, but her criticisms of poor William are tempered by womanly inconsistency. For instance she continues :—"William's present was finest of all, but I am hurt with him and myself, for he offered it so queerly . . . I refused it, and there's an end. 'Twas unmannerly and unkind of me and I have cried about it since."

The birthday entry contains a quaint description of a water party. "Father always gives me a birthday treat, so contrived that mother should take us to see my Lord Cardinal of York go to Westminster in state. We had a merry water party; got good places and saw the show, cross-bearers, pillar bearers, ushers and all, himself in crimson engrained satin and tippet of sables, with an orange in his hand held to his nose, as though the common air were too vile to breathe. What a pompous priest he is!" The day's festivities wound up with a family supper party. "After supper mother proposed a concert, and we were all singing round when looking up I saw father standing in the doorway with such a happy smile on his face. He was close behind Rupert and Daisy, who were singing together from the same book, and advertised his coming by gently knocking their heads together; but I had the first kiss, even before mother, as it was my birthday."

Following the birthday came very shortly a dialogue between the father and daughter on the subject of Margaret's betrothal to William Roper. She appears to have had literary aspirations, and had in consequence been sent by her father, who saw that her health was suffering from too prolonged application to study, to stay with some relatives in the country. "Or ever the week was gone father had contrived for me another journey to stay with some 'lay nuns,' as he called them, Aunt Nan and Aunt Fan, whom my stepmother loveth not, but whom I love and whom my father loveth. Indeed, 'tis said in Essex that at first he inclined to Aunt Nan rather than to my mother, but seeing that my mother affected his company and Aunt Nan affected it not he diverted his hesitating affections unto her and took her to wife. Of the sweet life of the "lay nuns" we read further on :—"How holy are my aunts' lives! Cloistered nuns could not be more sure and could scarce be as useful. Though wise they can be gay; though no longer young, they love the young; and their reward is the young love them, and I am full sure in this world they seek no better."

After her return from the visit to Essex, where her aunts were living, her father broached the subject of William Roper again and received no encouragement from his youthful daughter, who objected to the thought of losing her freedom, and was not inclined to William Roper as a husband at this period. Her objections were based on her observation of his lack of learning among other things and a want of facility in the expression of ideas with which she had little patience. Neither had her venerable father more patience with the freedom of her expression of these objections to an eligible suitor's advances. "'Thou art mad, my daughter, to look in a youth of Will's years for the mind of a man of fifty. 'But why need I concern myself about him?' I exclaimed; . . . 'I am young. I have much to learn, I love my studies; why interrupt them with other and less wise thoughts?'" Sir Thomas's reply is worthy of quotation, although it is in substance the summary of a philosophy not unheard before : "Because nothing is wise that is not practical, and I teach my children philosophy to fit them for living in the world and not above it. One may spend a life dreaming over Plato and yet go out without it, leaving the world a whit the better for our having part in it. . . . It is not necessary or good for us to live entirely with congenial spirits. The vigorous tempers the inert, the passionate is evened by the cool-tempered, the prosaic balances the visionary. . . . Even thine own sweet mother Meg was less affected to study than thou art. She learnt to love me for my sake, but I made her what she was." The parental counsel was not without its effect on the loving daughter, for she married William Roper and wondered how she formerly could have been senseless enough to have under-valued his sterling qualities. The Diary is continued up to the execution of Sir Thomas More, and in spite of inaccuracies and the fact that the style is far removed from sixteenth-century English it is a charming picture of a home life in which fiction and fact are blended with picturesque effect.

* "The Household of Sir Thomas More." By Anne Manning. (De la More Press. 1s. 6d. net.)

### Everybody's Opinion.

[Correspondence on all subjects is invited, but we cannot in any way be responsible for the opinions expressed by our correspondents. No communication can be entertained if the name and address of the correspondent are not given as a guarantee of good faith, but not necessarily for publication. All correspondents should write on one side of the paper only.]

### PROBATIONERS AND THEIR PLEASURES.

"Scottie" writes: Might I be allowed as an old Great Ormond Street probationer (now retired into private life) to offer a few remarks on the very "House Chatty" letter of the "Matron R. R. C." The whole gist of the matter lies in the words "I said nothing." Had the probationer gone to the matron herself at the time, instead of brooding over her grievances all those years, and laid bare her private affairs to the matron instead of years later to The Hospital I am sure the matron would have done her best for the probationer, and Henley might have been graced with the presence of the embryo "Matron R. R. C." My experience in that institution was very different. Knowing no one in London when my people did happen to come to town from the far north I found that by going direct to the matron I could make arrangements to spend my day off with them and these arrangements were never interfered with. "Hard" the matron might be, "unjust" never. But are days off not given more as a rest than for recreation? I have had both general and maternity training since I left Great Ormond Street, and in no hospital have probationers better off-duty times, and the arrangement which they had there, by which the humble probationer on her day off could have her breakfast in bed was so excellent that it might with advantage be copied by other matrons. Most of the Great Ormond Street nurses learned a lesson in etiquette which "Matron R. R. C." does not seem to have grasped—namely, never to take their old hospital's affairs into print unless they can say something good of it and of the management. It would be considered bad taste in a guest to make public the affairs of a house where she had been visiting, and the same holds good of a nurse and her training school. "Example is better than precept," we were told in our copy-books, and the writer of the letter in your last issue cannot expect her young nurses to be loyal to her if she is not equally loyal to her teachers in the past, even if their discipline was irksome.

### NURSEMAIDS AND UNIFORM.

"A Regular Reader" writes: I read with delight "A Trained Nurse's" letter *re* "Nursemaids and Uniform." I am not a hospital trained nurse yet, but I am selected for a vacancy next year in one of our largest hospitals; so I hope that I may be forgiven if I take up your valuable space. Some months ago I spent two weeks at Eastbourne, and was then disgusted to learn that so many girls, beautifully attired in perfect nurses' uniform, were after all only "nursemaids." On more than one occasion I noticed those "nurses" at nights sitting about the parade, walking, and even standing at corners, talking to boatmen, etc. Surely all nurses, who alone have the right to wear the modest uniform, should protest against this sort of thing. Would it be possible for hospitals to adopt a badge, which could be obtained only at the institutions by trained nurses? Or could not hospital nurses only be permitted to wear the long veil?

### ONLY A FEVER NURSE.

"R. M. C." writes: With your kind permission I should like to say a few words respecting the above. To become a good fever nurse long practice and study are necessary; yet, though many of us have passed through this course and made ourselves efficient, we are looked down upon as "only fever nurses," and, I think, are treated very unfairly. In 1892 I entered the largest fever hospital in England, went through my course of training, and obtained my certificate. Being devoted to the work, I decided to continue in it, and only procured a year of surgical nursing for the purpose of carrying it into my fever work if at any time I should require it. This is now my thirteenth year of fever nursing, during which time I have worked in our largest cities and in the worst epidemics of the country, including small-pox and typhus. Nevertheless I am asked to stand on one side for a nurse with a three years' certificate from a General Hospital who in nine cases out of ten has never seen small-pox, typhus, or a tracheotomy. If it rested with our medical men, I have good reason for believing that these things would not be allowed.

### "The Hospital" Convalescent Fund.

The Hon. Secretary begs to acknowledge with thanks the receipt of 7s. 6d. per the Travel Correspondent of The Hospital.

### Novelties for Nurses.

(By Our Shopping Correspondent.)

### THE CACHE HOLDFAST ENEMA.

The Holdfast Enema, sold by Messrs. W. H. Bailey and Son, 38 Oxford Street, W., is already well known, and is certainly the most convenient appliance of the kind. This excellent enema forms part of the "Cache Holdfast Enema." It is to be found in a small enamelled bowl enveloped in a case, which appears to be a sponge bag. Thus it is admirably suited for the use of travellers. It is conveniently packed and does not attract attention. It is also, of course, very suitable for general use.

### ADDRESSES.

The address of Egerton Burnett, Ltd., whose novelties in dress materials were noticed in this column last week, is The Royal Serge Warehouse, Wellington, Somersetshire; and that of Messrs. Brooks and Co., whose new catalogue was also noticed in the same number, 145, 147, 149 Borough High Street, London Bridge, S.E.

### TRAVEL NOTES AND QUERIES.

By our Travel Correspondent.

France in November (Autumn).—On the sum you propose it is impossible to spend a fortnight in France. Remember the cost of the journey. Brittany is the cheapest part of France in which to stay, but as your second-class return ticket to St. Malo would be £1 19s. 10d., you could not manage for so long a time, however reasonable your lodging might be. I suggest Belgium. Your journey by G.S.N.C. would be first return to Ostend 10s. 6d., second ditto 9s. Go from there to Bruges, a short journey, and only costing about 1s. 3d. each way. Bruges is full of interest. Write to Mrs. Dear, 25 Rue Cour de Gand, Bruges, Belgium, on a return prepaid foreign letter-card, and ask her lowest terms for this season and if she can take you in. Should her house be full, try Mademoiselle Boniface, 11 Quai au Miroir.

Holiday at Bruges (North).—The nurse did not give the address, and I do not know which convent she meant, but I will send you by post the address of one that I can recommend, and it is probably the same. Yes, French is generally spoken, except by the peasants, and they usually understand it, though they speak Flemish amongst themselves. You need no knowledge of Dutch at all. Bruges is in Belgium, not Holland, and if you cross the frontier you will find French or English will carry you far enough.

Rules in Regard to Correspondence for this Section.—All questioners must use a pseudonym for publication, but the communication must also bear the writer's own name and address as well, which will be regarded as confidential. All such communications to be addressed "Travel Correspondent, 28 Southampton Street, Strand." No charge will be made for inserting and answering questions in the inquiry column, and all will be answered in rotation as space permits. If an answer by letter is required, a stamped and addressed envelope must be enclosed, together with 2s. 6d., which fee will be devoted to the objects of "The Hospital" Convalescent Fund. Ten days must be allowed before an answer can be published.

### Notes and Queries.

**REGULATIONS.**

**The Editor is always willing to answer in this column, without any fee, all reasonable questions, as soon as possible.**

**But the following rules must be carefully observed.**

1. **Every communication must be accompanied by the name and address of the writer.**
2. **The question must always bear upon nursing, directly or indirectly.**

**If an answer is required by letter a fee of half-a-crown must be enclosed with the note containing the inquiry.**

*Carbolic Acid.*

(16A) Are carbolic crystals of practically the same strength as pure liquid carbolic, and how would one proceed to make a solution of 1 in 20?—*Inquirer.*

Carbolic acid, or phenol, in its pure state is in the form of colourless crystals. Liquid carbolic acid is prepared by the addition of 10 per cent. of water, and thus contains 90 per cent. of phenol; to make a solution of 1 in 20, mix 19 ounces of water with one ounce of the liquid carbolic acid. The slight error involved is negligable in practice.

*Nurses' Association.*

(17) Can you tell me if the —— Association is a good and reliable source to procure work from?—*C. F.*

We know nothing against the association you name.

*Training School.*

(17A) Will you please tell me if the — Hospital is a recognised training school for nurses?—*Inquirer.*

No, it does not at present fulfil the necessary conditions.

*The Up-Country Nursing Association.*

(18) Will you be good enough to tell me the address of the Secretary of the Up-Country Nursing Association? Is it a society for facilitating nurses going to foreign places under British rule?—*Nurse K.*

The Up-country Nursing Association is for Europeans in India, and the address of the Hon. Secretary is Dalkeith House, Cambridge Park, Twickenham.

*Unemployed Nurses.*

(19) Can you inform me where to apply for unemployed nurses?—*Slade.*

We are afraid that your only course is to advertise in the nursing papers or to answer advertisements.

*Probationer.*

(20) Can you let me know of any hospital where ladies of twenty-one are taken as probationers without paying a premium? I do not want a children's hospital.—*D. R. B.*

You might write to the Matron of the Penzance Infirmary and Dispensary, or answer advertisements.

*Phthisis.*

(22) I should feel very much obliged if you could kindly let me know through the columns of your paper if there is a home, or other place, at Davos, Switzerland, where a young gentleman suffering from phthisis could be taken for treatment at moderate terms? I have an idea that I have heard of a home there for English patients of limited means, opened in memory of our late Queen.—*K. O'C.*

The institution to be called "Queen Alexandra's Sanatorium" is not yet ready to be opened, and the Davos Invalids' Home is so small that it is almost impossible to obtain admission.

*District Nurse.*

(23) I should be much obliged if you would advise me in a small matter. I want to be a district nurse. I am trained.—*F. S.*

Write to the General Superintendent, Queen Victoria's Jubilee Institute for Nurses, 120 Victoria Street, London, S.W., if you think you would like to become a "Queen's" nurse. Otherwise write to some good association, such as the Shoreditch and Bethnal Green District Nursing Association, 80 Nichols Square, Hackney Road, London, N.E. Or advertise, or answer advertisements in our columns.

**Handbooks for Nurses.**

| | Post Free. |
|---|---|
| "A Handbook for Nurses." (Dr. J. K. Watson.) ... | 5s. 4d. |
| "Nurses' Pronouncing Dictionary of Medical Terms." ... | 2s. 0d. |
| "Art of Massage." (Creighton Hale.) ... ... ... ... | 6s. 0d. |
| "Surgical Bandaging and Dressings." (Johnson Smith.) | 2s. 0d. |
| "Hints on Tropical Fevers." (Sister Pollard.) ... ... | 1s. 8d. |

Of all booksellers or of The Scientific Press, Limited, 28 & 29 Southampton Street, Strand, London, W.C.

### For Reading to the Sick.

"HELP THOU MY UNBELIEF."

Lord, why then am I weak?—Because I give
Power to the weak, and bid the dying live.—

Lord, I am tired.—He hath not much desired
The goal, who at the starting-point is tired.—

Lord, dost Thou know?—I know what is in man;
What the flesh can, and what the spirit can.—

Lord, dost Thou care?—Yea, for thy gain or loss
So much I cared, it brought Me to the Cross.—

Lord, I believe; help Thou mine unbelief.—
Good is the word; but rise, for life is brief.

The follower is not greater than the Chief:
Follow thou Me along My way of grief.

*C. Rossetti.*

Lord, I believe, but would believe more fervently; O Lord, I love, but yet would love more warmly. I offer unto Thee my thoughts that they may be towards Thee; my deeds that they may be according to Thee; my sufferings that they may be for Thee.

*Anon.*

Often we cannot watch with Christ one hour. Our hearts are cold; our lips are stammering; our affections cleave to the dust; our spirits flag and tire at that which is at once angels' work and angels' rest. We can neither fix attention nor confine desire, nor realise God as present with us. Our thoughts set us at defiance, get entirely loose from our control.

See the disciples in that storm on the lake of Galilee, fearing for their lives though Christ was with them in the ship. See how gentle was the succeeding reproof, "Why are ye so fearful?" Jesus knew what was in man; and if in the hearts of His disciples at this time He saw but little faith, that little He knew was true. And so the merciful law was laid down for men of fearful heart, for all time, that God accepteth our faith, not for the measure of it, but for its sincerity.

Weak faith, mixed faith, little faith, better this than none at all. "Lord, I believe; help thou mine unbelief."

*Rev. D. Moore.*

"God with us": with us in every stage of our life; with us when we broke away; with us when we came back; with us as we were gaining strength, when the shorn locks of wilfulness began to grow again under returning faithfulness. "This God is our God for ever and ever: He shall be our Guide unto death."

So He restoreth my soul; so He leads me in the paths of righteousness; so He pledges to me the assurance of His Holy Name."

*Canon Randolph.*

And by the love that brought Thee here,
And by the Cross, and by the grave,
Give perfect love for conscious fear,
And in the day of Judgment save.
And lead us on, while here we stray,
And make us love our heavenly Home;
Till from our hearts we learn to say,
"Even so, Lord Jesus, quickly come."

*C. F. Alexander.*

